# Response to Antimony Toxicity in *Dittrichia viscosa* Plants: ROS, NO, H_2_S, and the Antioxidant System

**DOI:** 10.3390/antiox10111698

**Published:** 2021-10-27

**Authors:** Francisco Luis Espinosa-Vellarino, Inmaculada Garrido, Alfonso Ortega, Ilda Casimiro, Francisco Espinosa

**Affiliations:** Research Group of Physiology, Cellular and Molecular Biology of Plants, University of Extremadura, 06006 Badajoz, Spain; flespinosav@unex.es (F.L.E.-V.); igarridoc@unex.es (I.G.); a.ortega@enzazaden.es (A.O.); casimiro@unex.es (I.C.)

**Keywords:** antimony, AsA/GSH, *Dittrichia*, hydrogen sulfide, nitric oxide, reactive oxygen species

## Abstract

*Dittrichia viscosa* plants were grown hydroponically with different concentrations of Sb. There was preferential accumulation of Sb in roots. Fe and Cu decreased, while Mn decreased in roots but not in leaves. Chlorophyll content declined, but the carotenoid content increased, and photosynthetic efficiency was unaltered. O_2_^●−^ generation increased slightly, while lipid peroxidation increased only in roots. H_2_O_2_, NO, ONOO^−^, S-nitrosothiols, and H_2_S showed significant increases, and the enzymatic antioxidant system was altered. In roots, superoxide dismutase (SOD) and monodehydroascorbate reductase (MDAR) activities declined, dehydroscorbate reductase (DHAR) rose, and ascorbate peroxidase (APX), peroxidase (POX), and glutathione reductase (GR) were unaffected. In leaves, SOD and POX increased, MDAR decreased, and APX was unaltered, while GR increased. S-nitrosoglutathione reductase (GSNOR) and l-cysteine desulfhydrilase (l-DES) increased in activity, while glutathione S-transferase (GST) decreased in leaves but was enhanced in roots. Components of the AsA/GSH cycle decreased. The great capacity of *Dittrichia* roots to accumulate Sb is the reason for the differing behaviour observed in the enzymatic antioxidant systems of the two organs. Sb appears to act by binding to thiol groups, which can alter free GSH content and SOD and GST activities. The coniferyl alcohol peroxidase activity increased, possibly to lignify the roots’ cell walls. Sb altered the ROS balance, especially with respect to H_2_O_2_. This led to an increase in NO and H_2_S acting on the antioxidant system to limit that Sb-induced redox imbalance. The interaction NO, H_2_S and H_2_O_2_ appears key to the response to stress induced by Sb. The interaction between ROS, NO, and H_2_S appears to be involved in the response to Sb.

## 1. Introduction

Antimony (Sb) is a metalloid that is toxic for both plants and animals, including humans [1,2,3]. It is found at low concentrations in soils [4]. Nonetheless, its presence and polluting effects have been increasing considerably due to the growth in its mining and in industrial processes which use it, such as the manufacture of batteries [5,6,7,8]. It has recently been listed as a priority pollutant by both the European Union and the United States [9,10].

Sb is not an essential element for plants, but they can absorb and transport it [11]. Plants’ capacity to absorb this metalloid depends on which different species of Sb are in the soil and their bioavailability, as well as on the plant species itself [12,13,14]. If Sb absorption is high, toxicity problems result in reduced growth, impaired absorption of other mineral elements, slowed photosynthesis, development of oxidative stress, etc. [15,16,17,18,19].

As part of both normal physiological processes and processes under conditions of stress, different reactive species are produced in the various components of the plant’s cells. They include reactive oxygen, nitrogen, and sulfur species (ROS, RNS, and RSS, respectively) [20,21,22,23]. Thus, ROS and RNS are produced in the apoplast, cytoplasm, chloroplasts, mitochondria, and peroxisomes [22,24,25], and RSS in the cytoplasm, chloroplasts, and mitochondria [26].

Under stress conditions, plants’ levels of ROS (O_2_^●−^, H_2_O_2_, OH^−^, and ^1^O_2_) increase, leading to an imbalance in cellular redox homeostasis [27]. The RNS produced under these conditions include nitric oxide (NO), peroxynitrite (ONOO^−^), and S-nitrosoglutathione (GSNO) [28]. This increase in ROS and RNS production can trigger nitrosative stress [20,29,30]. The intervention of H_2_S and RSS in these processes has also been proven for different materials and stressors [31].

Heavy metal and metalloid toxicity induces a strong increase in the production of ROS, RNS, and RSS, which leads to alteration of the cellular redox balance [4,15,32,33,34,35,36,37,38,39]. Under stress conditions, the increase in ROS production leads to an alteration of the cellular redox balance [27]. As protection against the damage caused by the alteration of this redox balance and to eliminate the excess ROS, plants have developed an antioxidant system with both enzymatic and non-enzymatic components [21,40,41,42,43]. There also occurs an increase in the production of NO, which can react with ROS and thiol groups, regulating the expression of defence genes involved in the elimination of ROS, and re-establishing the redox balance [44,45,46]. In addition, the increased production of ROS and RNS and their interaction could act on the antioxidant systems involved in the response to heavy metals, enhancing their activity and the expression of genes involved in these antioxidant defence systems [30]. The interaction between H_2_S and NO in response to heavy metal stress [47,48] enables the effective elimination of ROS, with restoration of the redox balance. It has been shown that H_2_S has a stimulatory effect on the antioxidant system response [49] and on the gene expression of its components [50]. Furthermore, this interaction can modify proteins through persulfidation and S-nitrosation [51], thus altering their activity. Together, NO and H_2_S control the components of the AsA-GSH cycle and ROS levels, especially H_2_O_2_ [52]. The H_2_S and GSH relationship is especially important [53]. GSH shows a strong affinity to bind to heavy metals and metalloids, in addition to forming phytochelatins that can act by scavenging these elements. It has been shown for different plant species and for stress induced by various heavy metals [48,54,55] that the interaction between H_2_S and GSH reduces ROS production through induction of the antioxidant system. The interaction between ROS, NO, and H_2_S determines the tolerance to stress.

The objective of this investigation was to study the physiological response to Sb toxicity, shown by *Dittrichia viscosa*, a plant that accumulates this metalloid [56,57] and that is considered to be a good candidate for use in Sb, Cd, and As phyto-extraction processes [58,59]. In particular, we determined the effect that Sb toxicity has on biomass growth and production, mineral element absorption, photosynthetic pigment content, ROS, RNS, and H_2_S levels, and antioxidant systems.

## 2. Materials and Methods

### 2.1. Plant Materials, Growth Conditions, and Treatments

Seeds of *Dittrichia viscosa*, were surface sterilized for 15 min in 10% sodium hypochlorite solution (40 g L^−1^), rinsed several times with distilled water, and before their germination were imbibed in distilled water, aerated, and agitated for 2 h at room temperature. After imbibition, the seeds were germinated in a plastic container (30 cm × 20 cm × 10 cm) filled with a sterilized perlite mixture substrate wetted with a basal nutrient solution composed of 4 mM KNO_3_, 3 mM Ca(NO_3_)_2_ 4H_2_O, 2 mM MgSO_4_ 7H_2_O, 6 mM KH_2_PO_4_, 1 mM NaH_2_PO_4_ 2H_2_O, 10 μM ZnSO_4_ 7H_2_O, 2 μM MnCl_2_ 4H_2_O, 0.25 μM CuSO_4_ 5H_2_O, 0.1 μM Na_2_MoO_4_ 2H_2_O, 10 μMH_3_BO_3_, and 20 μM NaFeIII-EDTA, at 27 °C, in the dark, for 5 days. The seedlings were cultivated for 10 days at 27 °C with 85% relative humidity, and constant illumination under photosynthetic photon flux density (350 μmol m^−2^ s^−1^).

The seedlings grew for 10 days in hydroponic culture in lightweight polypropylene trays (30 cm × 20 cm × 10 cm; 4 plants per container) and the same environmental conditions (except for relative humidity, 50%). The plants were then treated with the basal nutrient solution supplemented with KSb(OH)_6_ to final concentrations of 0.00 (control), 0.50 mM, and 1.00 mM Sb. Each cultivation solution was adjusted to pH 5.8, continuously aerated, and changed every 4 days [17,38]. The plants were exposed to the Sb for 17 days. Plants of each treatment were divided into roots and shoots which were rinsed with distilled water, dried on filter paper, and weighed to obtain the fresh weight (FW). Half of the roots and shoots from each Sb treatment were dried in a forced-air oven at 70 °C for 24 h to obtain the dry weight (DW) and for the subsequent analysis of the concentration of Sb and other mineral elements. The other half of the roots and leaves were used for the biochemical assays.

### 2.2. Determination of Sb and Mineral Content

To determine the concentrations of Sb in the soil and leaves, the samples were maintained at 70 °C for 72 h, then crushed in a ceramic mortar. The assays were performed by inductively coupled plasma mass spectrometry (ICP-MS) [60], except for nitrogen which was determined by the Kjeldahl method [61].

### 2.3. Determination of Photosynthetic Pigment Contents and Photosynthetic Efficiency

Leaf discs from fresh adult leaves were taken and incubated in methanol for 24 h in darkness at room temperature. The chlorophyll a, chlorophyll b, and carotenoid contents were determined spectrophotometrically by measuring A_666_, A_653_, and A_470_. The total chlorophyll and carotenoid contents were calculated following Wellburn [62].

The maximum photosynthetic efficiency (F_v_/F_m_) was determined using a “ChlorophyllFluorometer OS-30p” device (Opti-Sciences). Prior to the excitation, the leaves of the plant being sampled were kept in darkness for 10 min, then illuminated for the emitted fluorescence to be measured and the F_v_/F_m_ value calculated [63].

### 2.4. Determination of Lipid Peroxidation and Reactive Oxygen Species (O_2_^●−^ and H_2_O_2_), NO and H_2_S Content

The peroxidation of membrane lipids was determined spectrophotometrically from the formation of MDA (malondialdehyde) from TBA (2-thiobarbituric acid). To this end, roots or leaves (0.1 g) were homogenized in 1 mL 0.25% TBA and 10% TCA (trichloroacetic acid), incubated at 95 °C for 30 min, filtered, and centrifuged at 8800× *g* for 10 min. The amount of MDA was determined from A_532_–A_600_ with extinction coefficient ε = 155 mM^−1^ cm^−1^, expressing the result as µmol MDA g^−1^ FW [64].

The production of O_2_^●−^ was measured from the formation of adrenochrome. Fresh roots or leaves (0.5 g) were homogenized at 4 °C in 1 mL 50 mM phosphate buffer (pH 6.0), 1 mM EDTA, 0.5 mM PMSF (phenylmethylsulfonyl fluoride), 1 mM β-mercaptoethanol, 1% PVPP. The homogenate was filtered and centrifuged at 39,000× *g* for 30 min at 4 °C, and the supernatant was used for the O_2_^●−^ determination. The production of O_2_^●−^ was measured from the oxidation of epinephrine to adrenochrome at A_480_ (ε = 4.02 mM^−1^ cm^−1^) in a reaction medium containing 1 mM epinephrine, 25 mM acetate buffer (pH 5.0), and the enzyme extract [65,66].

Hydrogen peroxide contents were determined in accordance with Sergiev et al. [67]. Briefly, fresh roots or leaves (0.5 g) were homogenized in ice bath with 5 mL 0.1% (*w:v*) TCA. The homogenate was centrifuged at 12,000× *g* for 15 min at 4 °C. Then 500 µL of the supernatant was added to 500 µL of 10 mM phosphate buffer (pH 7.0) and 1 mL 1 M KI. The A_390_ was recorded, and the H_2_O_2_ content was taken from a standard curve.

The NO was determined in accordance with Zhou et al. [68]. Fresh roots or leaves (0.5 g) were homogenized in 3 mL of ice-cold 50 mM acetic acid buffer (pH 3.6) containing 4% zinc acetate, using a mortar and pestle. The homogenate was centrifuged for 15 min at 11,500× *g* and 4 °C. The supernatant was collected and neutralized by the addition of 100 mg charcoal. After vortexing and filtration, the filtrate was leached and collected, and 1 mL was mixed with 1 mL of Griess reagent. The mixture was incubated for 30 min, and the A_540_ was measured. The NO content was estimated from the calibration curve using sodium nitrite as standard, and expressed as nmol g^−1^ FW.

For the determination of the H_2_S content, roots or leaves (1 g) were ground under liquid nitrogen and extracted by 1 mL 50 mM phosphate buffer (pH 6.8) containing 0.1 M EDTA and 0.2 M ascorbic acid. The homogenates were centrifuged at 14,000× *g* for 15 min at 4 °C, and 400 µL of the supernatant was injected into 200 µL 1% zinc acetate and 200 µL 1 N HCl, and incubated for 30 min. Then 100 µL 5 mM dimethyl-p-phenylenediamine dissolved in 7 mM HCl was added to the trap, followed by the injection of 100 µL 50 mM ferric ammonium sulfate in 200 mM HCl. After 15 min incubation at room temperature, the A_670_ was determined [69]. The content of H_2_S was estimated from the calibration curve using sodium sulfide as standard, and expressed as nmol g^−1^ FW.

### 2.5. Determination of Enzymatic Activities

Fresh roots or leaves (0.5 g) were homogenized at 4 °C in 1 mL 50 mM phosphate buffer (pH 6.0), 1 mM EDTA, 0.5 mM PMSF (phenylmethylsulfonyl fluoride), 1 mM β-mercaptoethanol, 1% PVPP. The homogenate was filtered and centrifuged at 39,000× *g* for 30 min at 4°C, and the supernatant was used for the enzyme assays. For the measurement of the MDAR activity, the fresh plant material was homogenized at 4 °C in 50 mM phosphate buffer (pH 7.8) and 1% PVPP. The homogenate was filtered and centrifuged at 20,000× *g* for 15 min at 4 °C, and the supernatant was used for the enzyme determinations. The protein content was determined by the Bradford method [70].

Superoxide dismutase (SOD, EC 1.15.1.1) activity was determined at A_560_ in a medium containing 50 mM phosphate buffer pH 7.8, 0.1 mM EDTA, 1.3 µM riboflavin, 13 mM methionine, and 63 µM 4-nitro blue tetrazolium (NBT), and the enzyme extract [71].

Ascorbate peroxidase (APX, EC 1.11.1.11) activity was determined by following the decrease in A_290_ for 3 min (ε = 2.8 mM^−1^ cm^−1^) in 0.1 M phosphate buffer (pH 7.5), 0.5 mM ascorbate, 0.2 mM H_2_O_2_, and the enzyme extract [72], expressing the result as µmol ascorbate min^−1^ mg^−1^ protein.

Peroxidase (POX, EC 1.11.1.7) activity was determined at A_590_ (ε = 47.6 mM^−1^ cm^−1^) [73] in 3.3 mM DMAB, 66.6 µM MBTH, and 50 mM phosphate buffer (pH 6.0) and the enzyme extract, and expressed as nmol DMAB-MBTH min^-1^ mg^−1^ protein.

Dehydroascorbate reductase (DHAR EC 1.6.4.2) activity was determined from the oxidation of DHA at A_265_ (ε = 14 mM^−1^ cm^−1^) [74] in a medium containing 0.1 M phosphate buffer (pH 6.5), 0.5 mM EDTA, 2.5 mM GSH, 0.5 mM DHA, and the enzyme extract. DHAR activity is expressed as nmol ascorbate min^-1^ mg^−1^ protein.

Monodehydroascorbate reductase (MDAR EC 1.6.5.4) activity was determined from oxidation of NADH at A_340_ (ε = 6.22 mM^−1^ cm^−1^) [75] in a medium containing 50 mM Tris-HCl buffer (pH 7.8), 10 mM AsA, 0.2 mM NADPH, 0.5 units of ascorbate oxidase, and the enzyme extract, expressing the result as µmol NADH min^−1^ mg^−1^ protein.

Glutathione reductase (GR EC 1.6.4.2) activity was determined at A_340_ from the oxidation of NADPH (ε = 6.22 mM^−1^ cm^−1^) [74] in a medium containing 0.1 M phosphate buffer (pH 7.5), 0.5 mM EDTA, 0.5 mM GSSG, 0.2 mM NADPH, and the enzyme extract, expressing the result as nmol NADPH min^−1^ mg^−1^ protein.

S-nitrosoglutathione reductase (GSNOR EC 1.2.1.46) activity was determined at A_340_ from the oxidation of NADH [76] in a reaction medium with 20 mM Tris-HCl (pH 8.0), 05 mM EDTA, 0.2 mM NADH, 400 µM GSNO, and the enzyme extract. GSNOR activity is expressed as nmol NADH min^−1^ mg^−1^ protein. 

Glutathione S-transferase (GST, EC 2.5.1.18) activity was determined spectrophotometrically at A_340_ for 6 min (1 min lag) in a reaction mixture with 0.1 M phosphate buffer (pH 7.0), 0.2 mM GSH, 0.1 mM CDNB (ε = 9.6 mM^−1^ cm^−1^) and the enzyme extract, and expressed as nmol CDNB min^-1^ mg^−1^ protein [77].

l-cysteine desulfhydrilase (l-DES) activity was measured in accordance with Riemenschneider et al. [78]. Briefly, the enzyme extract was obtained from 1 g of roots or leaves ground with a mortar and pestle under liquid nitrogen and homogenized in 1 mL of 20 mM Tris-HCl (pH 8.0). The homogenates were centrifuged at 15,000× *g* for 15 min at 4 °C. Then, L-DES activity was measured in 1 mL of 2.5 mM dithiothreitol, 0.8 mM l-Cys, 100 mM Tris-HCl (pH 9.0), and 0.1 mL enzyme extract. The addition of l-Cys initiates the reaction. After incubation for 15 min at 37 °C, the reaction was terminated with the addition of 100 μL of 30 mM FeCl_3_ in 1.2 N HCl and 100 μL of 20 mM N,N-dimethyl-p-phenylenediamine dihydrochloride in 7.2 N HCl. The formation of methylene blue was determined at A_670_, and the enzyme activity was estimated from the calibration curve using sodium sulfide as standard, and expressed as nmol H_2_S min^−1^ mg^−1^protein.

The coniferyl alcohol peroxidase (CA-POX) activity was recorded by measuring the decrease in absorbance as A_265_ in a reaction medium composed of 0.1 mM CA in 25 mM acetate buffer (pH 5.0) (ε = 7.5 mM^−1^ cm^−1^) and the enzyme extract. A unit of CA-POX is defined as the amount of enzyme required to cause the oxidation of 1 nmol CA per minute at 25 °C, pH 5.0.

Finally, for polyphenol oxidase (PPO, EC 1.14.18.1) activity, an enzyme extract was obtained from the fresh plant material (0.2 g) homogenized at 4 °C in 1 mL 50 mM phosphate buffer (pH 6.5) and 1% PVPP. The homogenate was filtered and centrifuged at 20,000× *g* for 130 min at 4 °C, and the supernatant was used for the PPO activity determination. PPO activity was determined by measuring A_390_ at 30 °C in a medium containing the enzyme extract, 100 mM phosphate buffer, Triton X-100, and 30 µM caffeic acid [79], expressing the result as U PPO mg^−1^ protein.

### 2.6. Determination of Phenolics Content

Phenols, flavonoids, and phenylpropanoid glycosides were extracted from fresh adult leaves (0.2 g) by homogenization in 1 mL methanol, chloroform, and 1% NaCl (1:1:0.5), filtering, and centrifuging at 3200× *g* for 10 min. Total phenols were determined spectrophotometrically at A_765_ with the Folin-Ciocalteu reagent [80], expressing the result as µg caffeic acid g^−1^ FW. Total flavonoid content was measured at A_560_ [81], expressing the result as µg of rutin g^−1^ FW.

### 2.7. Determination of the Components of the AsA/GSH Cycle

To determine the content of AsA, DHA, GSH, and GSSG, roots or leaves (1 g mL^−1^) were homogenized at 4 °C in 5% metaphosphoric acid in a porcelain mortar. The homogenate was filtered and centrifuged at 16,000× *g* for 20 min at 4 °C. The total ascorbate and glutathione assays were performed in accordance with De Pinto et al. [82]. In particular, the total ascorbate pool was determined in a reaction medium containing the extract, 150 mM phosphate buffer (pH 7.4), and 5 mM EDTA, which was incubated for 15 min in darkness. The result was then complemented with 0.5% NEM (N-ethylmaleimide), 10% TCA, 44% orthophosphoric acid, 4% dipyridyl, and 110 mM FeCl_3_, followed by incubation at 40 °C for 40 min in darkness. The reaction was halted with ice, and the A_525_ was measured. To determine the amount of AsA, 10 mM DTT (DL-dithiothreitol) was added to the reaction medium before incubation in darkness, while 100 µL of water was added to determine the ascorbate pool. The concentration of DHA was estimated from the difference between the total ascorbate pool (AsA + DHA) and AsA.

The total glutathione pool was determined by adding 0.4 µL of extract to 0.6 µL of 0.5 mM phosphate buffer (pH 7.5) [82]. The reaction medium containing the extract, 0.3 mM NADPH, 150 mM phosphate buffer (pH 7.4), 5 mM EDTA, and 0.6 mM DTNB (5,5’-dithiobis(2-nitrobenzoic acid)) was stirred for 4 min, 2 U mL^-1^ GR was added, and the A_412_ was measured. To determine the GSSG content, the mixture was incubated for 1 h in darkness with 2-vinylpyridine (20 µL) to eliminate GSH, and, to determine the glutathione pool, 20 µL of water was added. The amount of GSH was obtained as the difference between the total pool (GSH + GSSG) and the amount of GSSG.

### 2.8. Visualization and Determination of ROS, RNS, and H_2_S

The roots (20 mm) of control and Sb-treated plants were incubated for 30 min in the dark at 37 °C with either 25 µM DCF-DA (for H_2_O_2_ detection) or 10 µM DHE (for O_2_^●−^ detection) in 10 mM Tris-HCl, pH 7.4, then rinsed thrice for 15 min each time with the same buffer [83]. To determine NO and ONOO^−^, the roots were incubated for 60 min in the dark at 25 °C with 10 µM DAF-2DA (for NO detection) or 10 µM APF (for ONOO^−^detection) in 10 mM Tris HCl, pH 7.4. They were then rinsed thrice for 15 min each time with the same buffer [83]. To detect RSNOs, the intact root samples were incubated for 60 min in the dark at 25 °C with 10 mM NEM, rinsing thrice (for 15 min each time) with 10 mM Tris-HCl, pH 7.4. They were then incubated with 10 µM Alexa-Fluor 488 Hg-link phenylmercury for 60 min in the dark at 25 °C [84]. Finally, they were rinsed with the same buffer thrice (for 15 min each time). To detect H_2_S, the roots were incubated for 40 min with 100 µM 7-azido-4-methylcoumarin (AzMC) in 10 mM phosphate buffer (pH 7.4) in the dark and at room temperature [85], and then rinsed thrice with the same buffer (for 15 min each time). Finally, the whole (non-fixed) roots were sectioned, separating the apical (AZ) and elongation (EZ) zones, placing them on a slide and examining them under fluorescence microscopy (Axioplan-Zeiss microscope). In each case, λ_exc_ and λ_em_ were adjusted to the respective probe.

At least five roots were tested under each experimental condition and five independent repetitions were analysed. Images were processed and analyzed using the ImageJ program, and fluorescence intensity was expressed in arbitrary units.

### 2.9. Statistical Analyses

The data to be presented are the means ± SD of at least 10 replicates obtained from three independent experiments. Statistical analyses were performed using the Mann–Whitney U-test.

## 3. Results

### 3.1. Effect of Sb on the Growth of D. viscosa Plants

**Figure 1:**
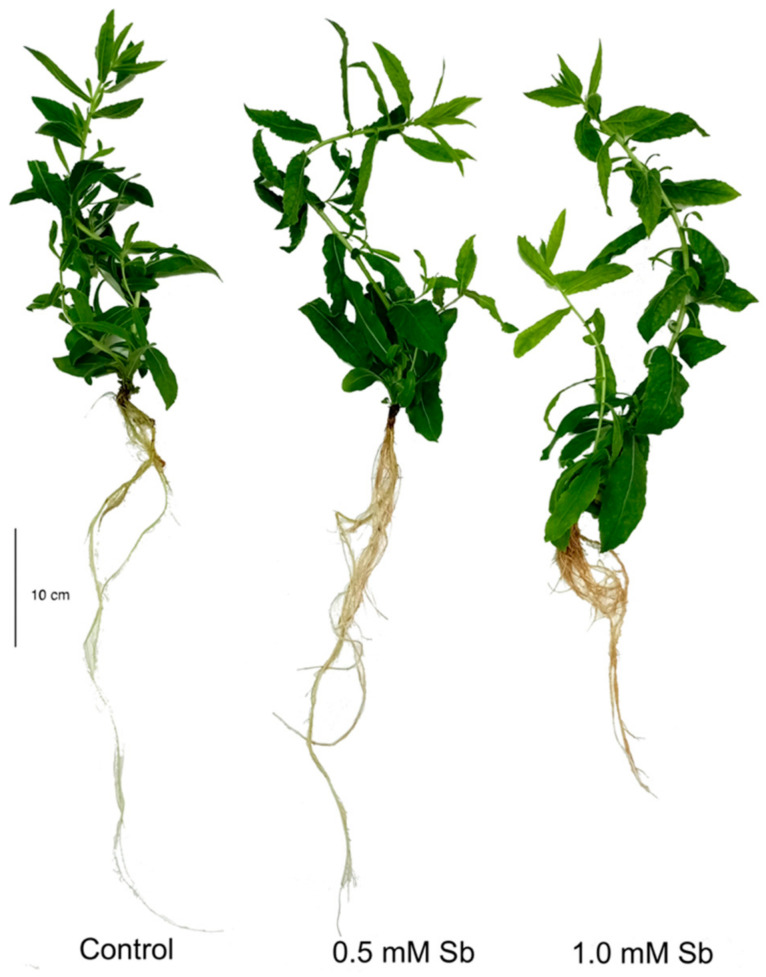
*Dittrichia viscosa* plants subjected to Sb toxicity for 17 days.

In the *D. viscosa* plants grown for 17 days (27 days of age) under toxicity of 0.5 mM and 1.0 mM Sb, a significant reduction in the length of both roots and stems was observed relative to the controls (Figure 1, Table 1). Thus, there were decreases of 28% and 36% in the root lengths under 0.5 mM and 1.0 mM Sb, respectively. The percentage decreases in stem lengths were less than those of the roots, being similar in value at 20% for both concentrations. There were reductions relative to the controls in the roots’ fresh and dry weights of 25% and 35%, and 33% and 45%, for 0.5 mM and 1.0 mM Sb, respectively. The same was the case for the stems, with decreases in the fresh and dry weights of 35% and 60%, and 17% and 39%, for 0.5 mM and 1.0 mM Sb, respectively. With respect to the total biomass produced, there were decreases relative to the controls of 25% and 50% for 0.5 mM and 1.0 mM Sb, respectively.

### 3.2. Effect of Sb on the Accumulation of Sb and Other Mineral Elements

The increase in the amount of Sb in the culture medium caused a significant and large increase in the absorption and accumulation of this element in both the roots and the leaves (Table 2). The increase in the amount of Sb in the medium causes increases in the content of this element, with the capacity for accumulation being greater in roots than in leaves. Thus, Sb causes an accumulation of this element in the roots of 13 503 µg g^−1^ FW in the presence of 0.5 mM, while if the concentration of the medium is 1.0 mM Sb then the accumulation reaches 24,450 µg g^−1^ FW. The amount of Sb that accumulates in the leaves is much lower, with values of 810 µg g^−1^ FW and 1547 µg g^−1^ FW, for 0.5 mM and 1.0 mM Sb, respectively. In the control roots, 4.1 µg g^−1^ FW was detected, and none was detected in the leaves. The bioaccumulation factor (BF) values clearly show the great capacity for absorbing and accumulating Sb in *D. viscosa* roots. The calculated BF values were similar for the two concentrations, although dependent on the organ. The absorption showed a tendency towards saturation, with very similar BF values. With respect to the transport capacity within the plant, the TF (translocation factor) values obtained were similar for the two concentrations (0.060 and 0.063, respectively).

The presence of Sb in the medium and its absorption by the tissues also altered the absorption and accumulation of other, essential, mineral elements. Table 3 lists their ionic concentrations in roots and leaves. Among the macronutrients, the N, S, and K content decreased in both roots and leaves to values that were very similar for the two Sb concentrations. The Ca and Mg content decreased only in the roots, remaining unaltered in the leaves. The exception among the macro-elements was P, which showed altered total content in neither roots nor leaves. With regard to micronutrients, the Fe and Cu contents underwent marked decreases, more so in roots than in leaves, and with the alteration in the Fe content being of greater magnitude than that of Cu. The Mn content in leaves remained unchanged, but decreased by 12% in roots for 1.0 mM Sb, but not for 0.5 mM Sb. The Zn content increased in roots for 0.5 mM Sb, but decreased (13%) for 1.0 mM Sb, while in leaves it decreased in a similar way for both concentrations relative to the control values. The B content declined in roots, but increased in leaves (20% for both toxic Sb concentrations).

### 3.3. Effect of Sb on Photosynthetic Pigment Content and Photosynthetic Efficiency

The data in Table 4 show how the 0.5 mM and 1.0 mM Sb toxicities produced a decrease of 15% and 36% in the chlorophyll a content and of 20% and 42% in chlorophyll b, respectively. Consequently, the total chlorophyll levels also decreased. The chlorophyll a/chlorophyll b ratio increased in response to Sb from 2.11 for the control to 2.35 for 1.0 mM Sb. The carotenoid content, however, increased in response to Sb stress, with similar increases of around 7% for the two concentrations of the metalloid. As a consequence of this increase in carotenoids and decrease in chlorophylls, the carotenoid/chlorophyll ratio increased, going from 0.056 for the control to 0.072 and 0.094 for the Sb treatments. The photosynthetic efficiency (EF) was unaltered by the 0.5 mM Sb treatment, but presented a slight decrease (approximately 10%) in the case of plants grown with 1.0 mM Sb. These results show an alteration in the chlorophyll and carotenoid contents in response to stress induced by Sb toxicity, although EF was only slightly affected by the higher concentration, reflecting the capacity of *D. viscosa* to maintain its levels of photosynthesis under these stress conditions.

### 3.4. Lipid Peroxidation and the Content of ROS, NO, H_2_S, ONOO^−^, and RSNOs

**Figure 2:**
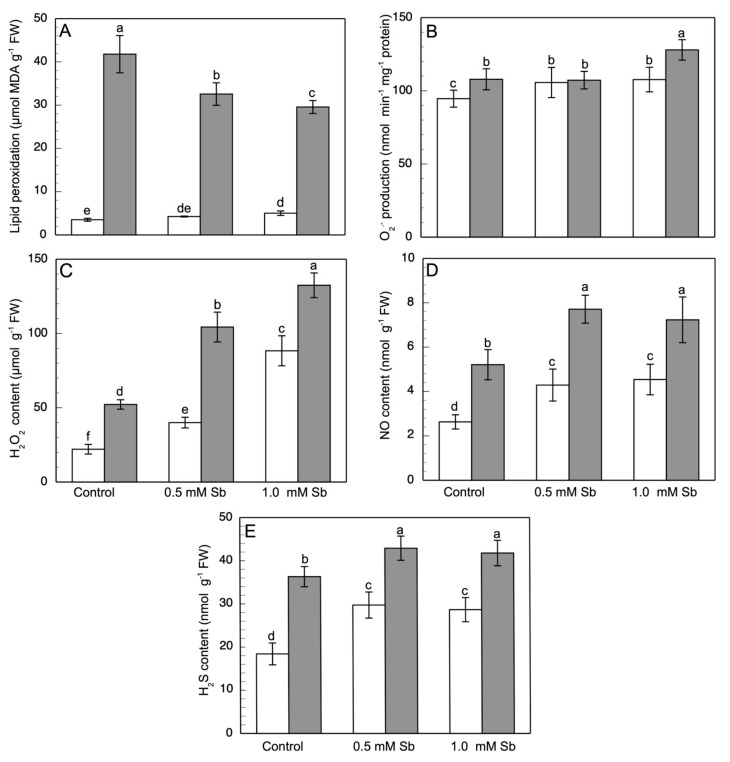
Effect of Sb on the lipid peroxidation (**A**), O_2_^−^ production (**B**), and H_2_O_2_ (**C**), NO (**D**) and H_2_S (**E**) content in roots (white) and leaves (gray) of *D. viscosa*. The data are from 10 independent experiments, each carried out in triplicate (different letters indicate significant differences at *p* < 0.05, Mann–Whitney U-test).

Oxidative damage was determined by the measurement of lipid peroxidation in both roots and leaves. In roots, Sb toxicity produced a strong increase in membrane lipid peroxidation, with increases of 21% and 43% for 0.5 mM and 1.0 mM Sb, respectively, compared to control values (Figure 2A). Surprisingly however, no increase was observed in lipid peroxidation levels in leaves, with values lower than the controls.

**Figure 3:**
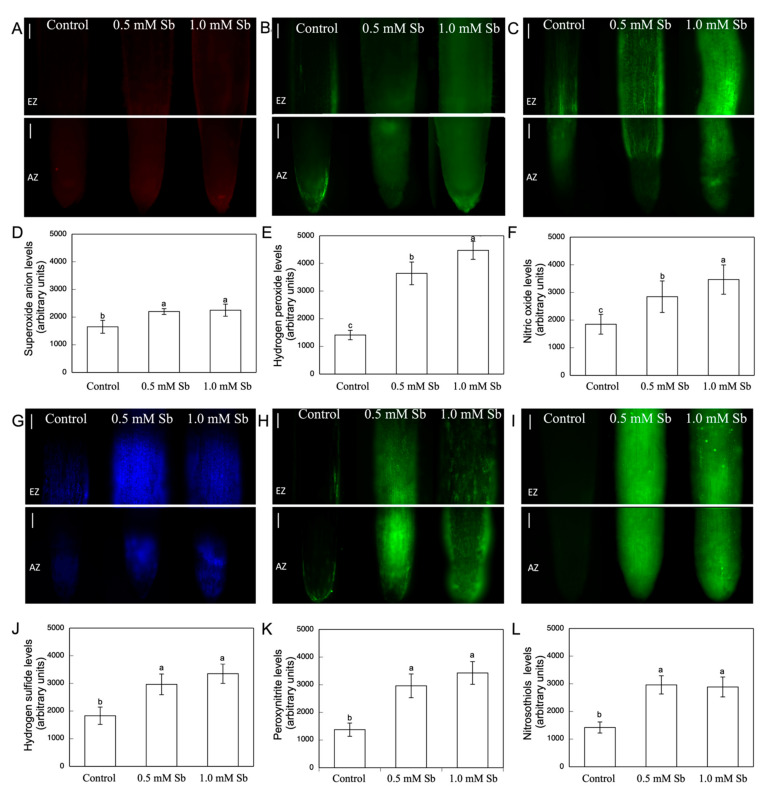
Detection of superoxide anion, hydrogen peroxide, nitric oxide, hydrogen sulfide, peroxynitrite, and S-nitrosothiols in the roots (**A**–**C**,**G**–**I**), and the average fluorescence intensity levels quantified in arbitrary units (**D**–**F**,**J**–**L**), respectively. At least five roots were tested for each experimental condition and five independent repeats were analysed (different letters indicate significant differences at *p* < 0.05, Mann–Whitney U-test). Bar: 200 μm.

With respect to the production of O_2_^●−^ (Figure 2B), there were increases in its content in roots that were similar in value (≈14%) for the two concentrations. In leaves, there was an 18% increase with 1.0 mM Sb, but no alteration for 0.5 mM Sb. These results coincide with the observation of the production and accumulation of O_2_^●−^ in roots using specific fluorescence probes (Figure 3A,D) in which one sees that the fluorescence is very similar in the roots treated with Sb, regardless of the concentration used.

The Sb toxicity caused a significant increase in H_2_O_2_ content in roots, by 82% and 346%, and leaves, by 100% and 153%, for 0.5 mM and 1.0 mM Sb, respectively (Figure 2C). With respect to the amount of accumulated H_2_O_2_ determined by fluorescence microscopy (Figure 3B,E), strong increases were observed in response to Sb toxicity (×2.5 and ×3.1 for the two concentrations of Sb).

The NO content under Sb stress conditions increased in both organs (Figure 2D), by 63.2% and 47.0%, and 72.3% and 38.0% for 0.5 mM and 1.0 mM Sb, in roots and leaves, respectively. The fluorescence images also show this clear increase in the NO content of the roots (Figure 3C,F), with increases in fluorescence levels very similar to the increases in NO content determined spectrophotometrically. 

Figure 2E shows the H_2_S content of roots and leaves of plants exposed to Sb toxicity. A strong increase was observed in the roots (52%, or ×1.5), being similar for the two Sb concentrations used. The behaviour was similar in the leaves, although the increase was more moderate (18%, ×1.2). Again, the fluorescence images allow this increase in the amount of H_2_S accumulated in the roots to be visualized (Figure 3G,J) (×2.2 and ×2.4 for 0.5 mM and 1.0 mM Sb, respectively).

The ONOO^−^ content also underwent a strong increase, as can be seen in both the fluorescence microscopy images (Figure 3H,K) (×2.2 and ×2.6, for 0.5 mM and 1.0 mM Sb). Sb also alters the S-nitrosothiol content (Figure 3I,L). As can be seen, there was a strong increase in the production and accumulation of these compounds for both concentrations of Sb used. Thus, the RSNOs increased by 52.9% when the roots were grown with 0.5 mM Sb, and 70.0% when they were grown with 1.0 mM Sb.

### 3.5. Effect of Sb on Enzymatic Activities

**Figure 4:**
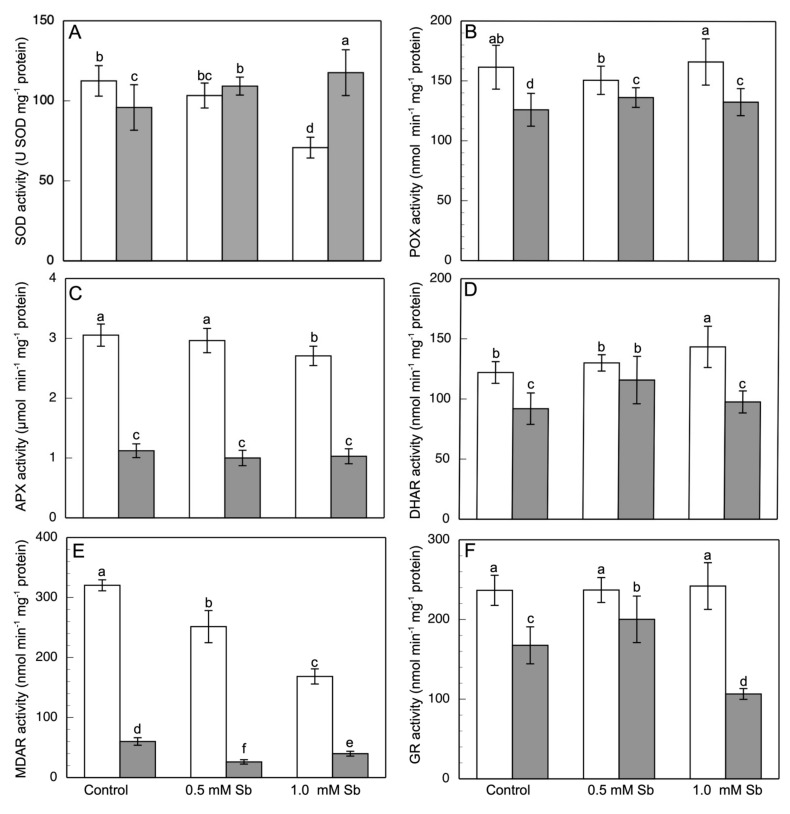
Effect of Sb on the SOD (**A**), POX (**B**), APX (**C**), DHAR (**D**), MDAR (**E**) and GR (**F**) activities, in roots (white) and leaves (gray) of *D. viscosa*. The data are from 10 independent experiments, each carried out in triplicate (different letters indicate significant differences at *p* < 0.05, Mann–Whitney U-test).

The SOD activity decreased in roots but increased in leaves in response to the Sb treatment (Figure 4A). Plants grown with 0.5 mM and 1.0 mM Sb showed decreases of 8% and 22%, respectively, in SOD activity in the roots, whereas in the leaves there were increases of 13% and 22.4%, respectively, relative to the values observed in control leaves. POX activity (Figure 4B) showed no alteration in roots, but slight increases in leaves. The APX activity (Figure 4C) in roots decreased by 11.5% under 1 mM Sb toxicity. In leaves, this decrease in APX activity was greater, by 10.5% and 8.4% for 0.5 mM and 1.0 mM Sb, respectively. DHAR activity (Figure 4D) in roots increased for both concentrations of Sb (15% for 1.0 mM Sb), whereas in leaves it increased for 0.5 mM Sb (12.5%) but not for 1.0 mM Sb. Nonetheless, MDAR activity showed decreases of 22% and 47%, and 56% and 44%, in roots and leaves, for 0.5 mM and 1.0 mM Sb, respectively (Figure 4E). With respect to the GR activity (Figure 4F), no changes were observed in roots treated with Sb, while in leaves the behaviour differed between the two concentrations, increasing by 19.7% for 0.5 mM Sb and decreasing by 36.5% for 1.0 mM Sb. 

**Figure 5:**
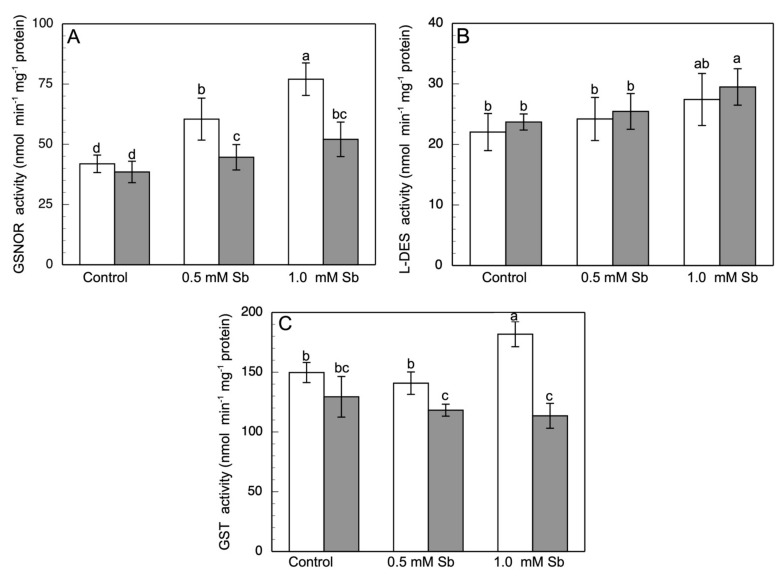
Effect of Sb on the GSNOR (**A**), L-DES (**B**) and GST (**C**) activities in roots (white) and leaves (gray) of *D. viscosa*. The data are from 10 independent experiments, each carried out in triplicate (different letters indicate significant differences at *p* < 0.05, Mann–Whitney U-test).

The GSNOR activity showed a strong Sb-induced increase of 44.4% and 83.8% in roots, and of 15.8% and 35.2% in leaves, for 0.5 mM and 1.0 mM Sb, respectively (Figure 5A).

With respect to the L-DES activity (Figure 5B), we detected an increase in roots, reaching values of 24.2 nmol min^−1^ mg^−1^ protein (10% increase) and 27.4 nmol min^−1^ mg^−1^ protein (24% increase) for 0.5 mM and 1 mM Sb, respectively. A similar behavior of this enzymatic activity was observed in leaves.

The GST activity in leaves showed decreases of 9% and 12% for 0.5 mM and 1.0 mM Sb, respectively. The behaviour in leaves was different since it was unaffected by 0.5 mM Sb, but under 1.0 mM Sb toxicity an increase of 21% was observed (Figure 5C).

**Figure 6:**
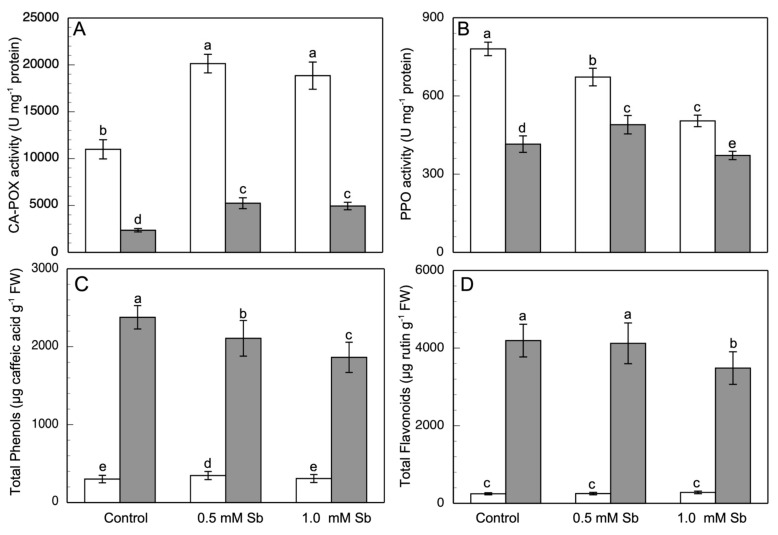
Effect of Sb on CA-POX (**A**) and PPO (**B**) and activities, and the total content of phenols (**C**) and flavonoids (**D**), in roots (white) and leaves (gray) of *D. viscosa*. The data are from 10 independent experiments, each carried out in triplicate (different letters indicate significant differences at *p* < 0.05, Mann–Whitney U-test).

Finally, CA-POX was the activity, Figure 6A, most strongly affected by the Sb toxicity. This activity increased by 83.1% and 71.2% in roots, and 122.5% and 109.0% in leaves, for 0.5 mM and 1.0 mM Sb, respectively.

With respect to the PPO activity (Figure 6B), in roots it decreased, with a dependence on the Sb concentration (14.3% and 35.4%, respectively). In leaves, PPO showed a similar behaviour at 1.0 mM Sb, but with 0.5 mM Sb there was an increase in this activity (15.4%).

### 3.6. Effect of Sb on the Phenolic Compound Content

The total content of phenolics, (Figure 6C) increased in roots of the 0.5 mM Sb treatment, while in leaves this content decreased (by 11.5% and 21.8%, for 0.5 mM and 1.0 mM Sb). A similar response was found for the contents of flavonoids (Figure 6D).

### 3.7. Effect of Sb on the Components of the AsA/GSH Cycle

In roots, the AsA content showed a reduction that ranged from 8.5% (0.5 mM Sb) to 16.1% (1.0 mM Sb) (Table 5). On the contrary, in leaves the two Sb treatments produced a very similar increase in this content. With respect to the DHA content, in the roots this increased for 0.5 mM Sb but decreased when the Sb concentration was 1.0 mM. On the contrary, the DHA content in leaves decreased for both concentrations (30.1% and 54.5%, respectively). These alterations cause the total AsA + DHA content to decrease in both roots and leaves except for 0.5 mM Sb in roots when there was no alteration. Finally, the AsA/DHA ratio showed a decrease in roots for 1.0 mM Sb but not for 0.5 mM Sb, and an increase in leaves (×2 for 1.0 mM Sb).

## 4. Discussion

Similar to the toxicity due to other heavy metals and metalloids such as Cd, Pb, and Ni [42,54,86,87] the stress induced by high concentrations of Sb in the medium reduces growth and biomass production in *Dittrichia viscosa*. Exposure of *D. viscosa* plants to 0.5 mM and 1.0 mM Sb for 17 days reduced both the length and the fresh and dry weights of the roots and stems, as well as the total biomass production. This effect was clearly dependent on the Sb concentration. The growth inhibition effect was more pronounced in the roots than in the aerial part. These results are similar to those described by other workers for other plants under toxicity from this metalloid [4,15,17,38,88].

As described above, the amount of Sb accumulated more in roots than in leaves, with this accumulation being dependent on the amount and form of Sb in the medium [18,37,89,90]. The values of BF of roots were much greater than those of leaves. The low TF value obtained in *D. viscosa* contrasts with the values reported by Benhamdi et al. [91] of 1.21 and 0.34 for *H. pallitum* and *L. spartum*, respectively. Nonetheless, these low TF values coincide with those observed by Pérez-Sirvent et al. [92] also in *D. viscosa* at different mining locations, although with lower accumulation levels, possibly due to the lower amount of Sb available in the soils they studied compared to the levels provided in the present work.

The absorption and transport of other mineral elements was also affected by the Sb toxicity. The presence of high amounts of Sb in the medium caused the content of N, S, K, and Ca to decrease in both roots and leaves. Nonetheless, the P content was unaltered. Shtangeeva et al. [93] describe a decrease in Ca and K content under Sb toxicity, while Zhu et al. [14] describe the decrease in K content but an increase in Ca content. Mg content decreases in roots [14,17], but the decrease was much lower in leaves, observing a TF for this element much greater than that of the control. It is possible that this factor could be related to the maintenance of photosynthetic pigment levels that can maintain EF under these toxic conditions, especially in the case of 0.5 mM Sb. The decrease in Fe and Cu content was clear and coincides with the decreases observed by other workers [1,14,94]. In tomato, Sb induces a decrease in Fe and Zn content, with increases in Mg and Cu [38]. The behaviour of Mn was different. It declined in roots, as also observed by Zhu et al. [14] in rice and by Espinosa-Vellarino et al. [17] in tomato, but it remained basically unchanged in leaves, with just slight fluctuations, and this, together with the lower decrease in the Mg content of leaves, might contribute to maintaining the EF. This result is similar to that described by Rodríguez-Ruíz et al. [95] who observed decreases in the content of Mg and Mn in roots but not in leaves of pea plants subjected to As toxicity.

The chlorophyll content decreased in response to Sb toxicity, due either to a decrease in its biosynthesis [33,96,97] or to an increase in its degradation [98,99]. The chlorophyll a/b ratio increased in response to treatment with Sb, indicative of a response to metalloid toxicity as described by other workers [88]. This increase is due to the greater decrease in chlorophyll b content than that observed in chlorophyll a, possibly because of a greater degradation of chlorophyll b than chlorophyll a [98,99]. Nonetheless, the carotenoid content increased, which reflects its antioxidant and photoprotective capacity [100], while in *A. calamus* under Sb toxicity, a decrease in both the carotenoid content and the carotenoid/chlorophylls ratio has been observed [88]. This increase in the carotenoid content causes the carotenoid/chlorophyll ratio to also increase, due to both the increase in carotenoids and the decrease in the total chlorophyll content. Sb toxicity induced an increase in ROS production that affects negatively the chlorophyll content. The chlorophyll a/b ratio is related to the degree of capture and fluidity in the thylakoid membranes [101,102] in such a way that the greater the ratio, the lesser the capture and also the lesser the photosynthetic capacity [33,88]. In previous research by our group [17,38], we observed in sunflower and tomato how Sb induces an increase in the chlorophyll a/b ratio, which leads to decreases in EF. However, surprisingly, *D. viscosa* maintains EF levels under Sb toxicity similar to the control values. A slight and non-significant decrease was observed only with 1.0 mM Sb. The increase in this ratio in *D. viscosa* is percentage-wise less than that observed in sunflower and tomato [17,38]. These data reflect an efficient operation of photosystem II even in these conditions of high Sb toxicity. This result contrasts with those obtained by other workers [33,96] who observed, together with the decrease in chlorophylls, an increase in the energy dissipation flow, which leads to a decrease in EF in response to Sb toxicity. In *D. viscosa*, the F_0_ and F_m_ values remained constant, which makes the EF do so as well. The stable maintenance of F_0_ values may indicate that the PSII reaction centre is unaltered, with no reduction of the energy flow from the antenna chlorophylls to the PSII. This effect may be due to the control in the production of ROS. Furthermore, the unchanged F_m_ seems to indicate that neither are there any significant alterations in the ultrastructure of the thylakoid membranes, despite their impaired stacking, that would be able to negatively affect the electron flow. It is possible that the increase in carotenoid content may help maintain the EF. Carotenoids can act by eliminating ROS, thereby protecting the PSII functionality from oxidative damage [100,103,104,105].

In roots, treatment with Sb induced an increase in the content of H_2_O_2_ and, to a lesser degree, of O_2_^−^. A consequence of this increase in ROS is the increase in lipid peroxidation. Nonetheless, in leaves no increase was observed in lipid peroxidation despite the increase in O_2_^−^ and H_2_O_2_ production. The increase in the production of ROS induced by Sb in *D. viscosa* is similar to that described for other plants in response to toxicity from both Sb and other heavy metals [17,35,38,55,57,87,106,107,108]. Nonetheless, the increase in O_2_^●−^ production was much less than that observed previously by our group for sunflower and tomato [17,38], reflecting *D. viscosa*’s greater tolerance to Sb stress. In plants subjected to Cd toxicity, increases are observed in both lipid peroxidation and H_2_O_2_ content [55,87]. The increase in lipid peroxidation in response to stress from Sb and heavy metals has also been observed in different plants [15,17,38,97]. The non-alteration of lipid peroxidation levels in the leaves may be related to the efficacious performance of the antioxidant system and to the ability to retain most of the Sb in the roots (TF ≈ 0.06). Other workers [109,110] have also observed that Sb has a low impact on lipid peroxidation.

The slight increase in the production of O_2_^−^ is in line with the decrease in SOD activity observed in roots and the small increase in leaves. The enzymatic antioxidant system is altered by the Sb toxicity. The POX and APX activities behaved similarly in roots and leaves, not increasing but presenting values either similar to the controls or lower. The decrease in these activities causes accumulation of H_2_O_2_, despite the decreased SOD activity in roots but not in leaves. On the contrary, numerous studies have observed an increase in these activities in response to Sb [17,32,38,91,111]. Nonetheless, in *Dittrichia*, a plant tolerant to Sb [56,57,94], the SOD and APX activities decrease in roots. The different responses of these antioxidant enzymes depending on the organ was also observed by Rodríguez-Ruíz et al. [95] for pea plants subjected to As stress, for which the expression of SOD decreased strongly in roots but not in leaves, while APX activity increased in roots and decreased in leaves. The increase in H_2_O_2_ content could be being used for lignification processes that tend to reduce the entrance of Sb into the roots. This is supported by the strong increase shown by CA-POX (apoplastic Class III peroxidase) activity in response to Sb toxicity. Furthermore, although the total phenolic compound content was unaltered, there was a decrease in PPO activity. This decrease may help maintain the total content of polyphenols, flavonoids, and other phenolic compounds with strong antioxidant activities. Furthermore, by oxidizing phenols, CA-peroxidase can give rise to the development of a barrier in cell walls which reduces the entry and immobilization of Sb [111,112,113].

The DHAR activity increased in roots, while in leaves it increased for 0.5 mM Sb but remained unchanged for 1.0 mM Sb. MDAR activity decreased in both organs, and GR activity showed no significant changes in roots but in leaves increased for the 0.5 mM Sb treatment and decreased for 1.0 mM Sb. These activities thus showed changes in their behaviour that were dependent on the organ studied. Singh et al. [114], for luffa leaves subjected to As toxicity, describe an increase in GR activity and decrease in DHAR and MDAR activities. Rodríguez-Ruíz et al. [95] describe increases or decreases in organ-dependent GR, MDAR, and DHAR activities in response to toxicity of this same heavy metal. On the contrary, Feng et al. [32] describe a decrease in GR activity induced by Sb toxicity. For tomato subjected to Sb toxicity, there is no modification in either DHAR or GR activity [17]. The decrease in GST activity in leaves observed in the present study may be related to the lesser decrease in GSH content that we observed in leaves compared to roots. In addition, the GSH/GSSG ratio declined less in leaves than in roots. Zhang et al. [115] observe in rice subjected to Cd toxicity an organ-dependent behaviour of GST, with increases in leaves and decreases in roots, suggesting the possible existence of different isoenzymes and their expression. Mostofa et al. [54] observe a decrease in GST activity in leaves of rice subjected to Cd toxicity, with increases in the rest of the antioxidant enzymatic activities. The increased activity observed in roots under Sb toxicity coincides with the results of Benhamdi et al. [91] who describe increases in GST activity under Sb and As toxicity, in their case in both roots and leaves. Hou et al. [116] observe in *Arabidopsis thaliana* that the presence of Cd alters the protein structure of GST, reducing its activity and thereby affecting the antioxidant system. In *Dittrichia* roots, the high amount of Sb induced GST, which also may bind directly to Sb. In leaves, the lower concentration of Sb may be detoxified by direct interaction with GSH.

Sb acted on NO metabolism, producing an increase in both the NO and ONOO^−^ contents, with an increase in GSNOR activity as well. The increase in these compounds and GSNOR was stronger in roots than in leaves. These results coincide with those obtained by Leterrier et al. [117] for *Arabidopsis* under As stress, with increased NO and GSNOR, and decreased GSH. In addition, for sunflower and tomato [17,37] Sb induces increases in the content of NO, ONOO, and GSNOR activity, although these activating effects vary depending on the organ. On the contrary, Rodríguez-Ruíz et al. [95] describe for peas with low As toxicity an inhibitory effect on both the GSNOR activity and the formation of NO and ONOO.

With respect to the effect of H_2_S, most works [51,108,114,118,119] have studied its exogenous application, finding it to act by enhancing the antioxidant system, and thereby, improving the defence against heavy metal toxicity. The interconnection between H_2_S and NO is evident in a large number of studies [26,120,121]. In *Dittrichia*, Sb toxicity induced an increase in L-DES activity, which also entailed an increase in H_2_S content, more marked in roots than in leaves. This increase in activity may be related to the increase in NO content [122] which is observed under Sb toxicity. There are no references in the literature to the effect of Sb toxicity on the content and functionality of endogenous H_2_S. Increases in the content of H_2_S and NO have also been described by Kaya et al. [55] for Cd toxicity in wheat, with a notable increase in H_2_O_2_ content and SOD and POX activities. In *Isatis indigotica*, Jia et al. [48] observe plants under Cd toxicity to show an increase in endogenous H_2_S, which induces the formation of Cd-chelating compounds in addition to acting by decreasing the flow of Cd through the membranes. On the contrary, Kushwaha and Singh [49] find a decrease of the effects of Cr toxicity on endogenous H_2_S levels, although the application of exogenous H_2_S acts to mitigate the effects of the oxidative stress induced by the heavy metal by boosting the antioxidant response.

In *Dittrichia*, the increases we observed in the NO and H_2_S content may act to improve the plant’s response to the toxicity of this heavy element [123,124,125]. The increase in the content of NO may contribute in *Dittrichia* to reducing both the formation of O_2_^●−^, through its inhibitory effect on RBOHD, and its accumulation, through the formation of ONOO. The increase in GSNOR activity would contribute to maintaining the level of NO, thus regulating its effects.

Sb altered the content of the components of the AsA/GSH cycle. GSH plays an important role in maintaining cellular redox homeostasis [126,127]. Our results showed that, in *Dittrichia*, Sb induces a decrease in AsA content in roots but not in leaves, whereas DHA decreases in both organs. This alteration might be related to the alterations we observed in APX, DHAR, MDAR, and GR activities. While AsA/DHA declined slightly in roots, it rose in leaves. In tomato roots exposed to Cr [128], the AsA content decreases, without significant changes in DHA, leading to a decrease in AsA/DHA.

Sb also caused decreases in the GSH and GSSG contents and the GSG/GSSG ratio, especially in the roots. Such alterations in the GSH and GSSG contents and the GSH/GSSG ratio have been described by other workers. Thus, Kushwaha et al. [128] for Cr toxicity in tomato roots, and Cui et al. [129] and Mostofa et al. [54] for Cd toxicity in alfalfa roots and leaves and rice leaves, respectively, report similar decreases in GSH content and GSH/GSSG. In addition, for peas under As toxicity, Rodríguez-Ruíz et al. [95] describe decreases in the GSH and GSSG content in both roots and leaves. The decrease in GSH content may be due to its capacity to serve as a substrate for phytochelatins [95] which would subsequently act to chelate Sb, or to Sb’s capacity to bind either directly to GSH or through GST activity [130]. The greater decline in GSH content together with the increased GST activity observed in roots may, together with the strong increase in the coniferyl alcohol peroxidase activity, be the cause of the large accumulation of Sb that occurred in this organ.

Sb has a high capacity to bind thiol groups [131]. This formation of ligands may explain the decrease observed in the GSH and GSSG content and GSH/GSSG. This effect was stronger in roots, where the greatest accumulation of Sb is found. In addition, the decrease in GSH levels migth also be correlated with the use of this compound for the synthesis of phytochelatins capable of forming complexes with Sb [95,132].

Sb toxicity causes an increase in the amount of thiols, thus favouring its immobilization, and reducing its absorption and transport [122], as was the case with the low TF in *Dittrichia*.

Shi et al. [133] show the necessary H_2_S and NO interaction in the response to heavy metals. The increase in the H_2_S and NO content under conditions of Sb toxicity causes an increase in the nitrosothiol content, compounds which help boost the defence system [134]. Levels of endogenous H_2_S and NO increase in response to treatment with Cd [121]. NO can prevent increases in O_2_^●−^ and lipid peroxidation [133,135]. It acts by maintaining the stability of the electron transport systems, and consequently PSII activity [125]. The increase in H_2_S, in addition to its interaction with NO and H_2_O_2_, can also act to maintain photosynthetic efficiency [136], and affect both antioxidant enzymes and AsA and GSH to keep ROS levels low [51].

## 5. Conclusions

In *Dittrichia viscosa*, treatment with these concentrations of Sb induced nitro-oxidative stress in both roots and leaves. The great capacity of *Dittrichia* roots to accumulate Sb is the reason for the differing behaviour observed in the enzymatic antioxidant systems of the two organs. In roots, the increase in ROS levels induced by Sb was correlated with an increase in the levels of lipid peroxidation, reflecting the development of oxidative damage at the level of this organ. It was in the roots where the greatest amount of Sb was detected in the plant, with little capacity for translocation to the aerial part. Sb toxicity caused alterations in SOD, APX, DHAR, MDAR, and GR activities in roots, but little alteration in leaves. The components of the AsA/GSH cycle decreased, which, together with the increase in GST activity in roots, shows the priority effect Sb has on GSH, binding to it, with this being the main form of accumulation and detoxification that *Dittrichia* presents in response to this metalloid. Sb altered the ROS balance, especially with respect to H_2_O_2_. This led to an increase in NO and H_2_S acting on the antioxidant system to limit that Sb-induced redox imbalance. In addition, the increases in NO, ONOO^−^, and nitrosothiol content and GSNOR activity are evidence for the participation of RNS in this response. Possibly, most of the excess H_2_O_2_ might be used in the process of lignin synthesis in both root and leaf cell walls as an immobilization system.

## Figures and Tables

**Table 1:** Effect of Sb on the length, fresh weight, and dry weight of roots and stems, and on total biomass production in *D. viscosa* plants. The data are from 10 independent experiments, each carried out in triplicate (different letters indicate significant differences at *p* < 0.05, Mann–Whitney U-test).

Treatments	Length (cm)		FW (mg)		DW (mg)		Biomass Production (%)
	Roots	Stems	Roots	Stems	Roots	Stems	
Control	47.11 ± 1.95a	29.41 ± 1.05a	3460 ± 210a	5930 ± 243a	232 ± 18a	817 ± 51a	100a
0.5 mM Sb	34.32± 1.11b	23.43 ± 1.15b	2620 ± 200b	3853 ± 240b	167 ± 13b	687 ± 39b	75.5 ± 3.1b
1.0 mM Sb	29.68 ± 1.50c	24.13 ± 0.66b	2250 ± 200c	2404 ± 87c	126 ± 14c	506 ± 25c	47.0 ± 2.8c

**Table 2:** Sb content in roots and leaves, bioaccumulation factor (BF), and translocation factor (TF) in *D. viscosa* plants. The data are from 10 independent experiments, each carried out in triplicate (different letters indicate significant differences at *p* < 0.05, Mann–Whitney U-test).

Treatments	Sb (µg Sb g^−1^ FW)		BF		TF
	Roots	Leaves	Roots	Leaves	
Control	4.1 ± 1.1c	--	--	--	--
0.5 mM Sb	13,503 ± 225b	810 ± 52.5b	224a	12.1a	0.060a
1.0 mM Sb	24,450 ± 356a	1547 ± 75.0a	202a	12.8a	0.063a

BF: the ratio between the concentration of element in the roots or leaaves and that present in the soil.TF: the ratio between the concentration of element in the leaves and in the roots.

**Table 3:** Effect of Sb on the mineral element content of roots and leaves of *D. viscosa* plants. The data are from 10 independent experiments, each carried out in triplicate (different letters indicate significant differences at *p* < 0.05, Mann–Whitney U-test).

Treatments	N (mg g^−1^ DW)		P (mg g^−1^ DW)		S (mg g^−1^ DW)		K (mg g^−1^ DW)		Ca (mg g^−1^ DW)		Mg (mg g^−1^ DW)	
	Roots	Leaves	Roots	Leaves	Roots	Leaves	Roots	Leaves	Roots	Leaves	Roots	Leaves
Control	38.0 ± 0.45a	39.2 ± 5.1a	37.6 ± 4.0a	32.3 ± 3.8a	7.5 ± 1.0a	4.4 ± 0.9a	43.2 ± 5.0a	39.0 ± 5.8a	20.5 ± 3.0a	17.4 ± 2.5a	9.7 ± 1.5a	7.2 ± 1.1a
0.5 mM Sb	27.4 ± 0.38b	28.8 ± 4.0b	38.6 ± 4.1a	32.4 ± 4.2a	4.9 ± 1.2b	3.7 ± 0.7a	31.0 ± 4.5b	28.9 ± 3.3b	14.1 ± 2.2b	17.0 ± 1.9a	4.7 ± 0.8b	6.0 ± 1.5a
1.0 mM Sb	26.9 ± 0.30b	23.4 ± 3.3b	37.2 ± 4.7a	30.8 ± 5.0a	5.2 ± 1.1b	3.2 ± 0.9b	31.5 ± 4.b1	27.4 ± 3.5b	13.9 ± 2.7b	16.3 ± 2.7a	4.6 ± 1.0b	6.1 ± 1.3a
**Treatments**	**Fe (μg g^−1^ DW)**			**Mn (μg g^−1^ DW)**			**Cu (μg g^−1^ DW)**		**Zn (μg g^−1^ DW)**		**B (μg g^−1^ DW)**	
	**roots**	**leaves**		**roots**	**leaves**		**roots**	**leaves**	**roots**	**leaves**	**roots**	**leaves**
Control	2298.3 ± 176.4a	631.4 ± 81.4a		753.3 ± 60.5a	245.5 ± 31.3a		94.8 ± 10.1a	63.9 ± 6.1a	55.4 ± 6.2b	78.0 ± 8.5a	165.2 ± 19.8a	239.2 ± 18.5b
0.5 mM Sb	878.6 ± 22.6b	526.7 ± 64.5b		769.2 ± 91.5b	260.6 ± 33.5a		39.6 ± 4.2c	55.8 ± 6.2b	80.2 ± 7.4a	64.4 ± 5.7b	123.6 ± 13.4b	288.0 ± 25.5a
1.0 mM Sb	620.9 ± 68.5c	469.0 ± 55.3b		440.6 ± 52.8c	208.8 ± 34.0b		49.1 ± 5.3b	44.5 ± 5.6c	48.5 ± 5.8b	63.5 ± 5.1b	56.9 ± 5.8c	285.4 ± 25.9a

**Table 4:** Effect of Sb on the chlorophyll a and b and total chlorophyll contents (µg g^−1^ FW), chlorophyll a/b ratio, total carotenoids (Car) (µg g^−1^ FW), carotenoid/chlorophyll ratio, and photosynthetic efficiency (F_v_/F_m_) in *D. viscosa* leaves. The data are from 10 independent experiments, each carried out in triplicate (different letters indicate significant differences at *p* < 0.05, Mann–Whitney U-test).

Treatments	Chl a (μg g^−1^ FW)	Chl b (μg g^−1^FW)	Chl a+b (μg g^−1^ FW)	Chl a/chl b	Carotenoids (μg g^−1^ FW)	Carotenoids/Total chl	Fv/Fm
Control	1773.5 ± 42.6a	838.7 ±46.4a	2617.8a	2.11c	145.9 ± 7.1b	0.056c	0.789 ± 0.018a
0.5 mM Sb	1512.8 ±57.2b	672.1 ± 34.5b	2188.3b	2.26b	157.5 ± 6.3a	0.072b	0.783 ± 0.026a
1.0 mM Sb	1144.0 ± 34.1c	488.2 ± 28.6c	1630.8c	2.35a	155.1 ± 7.4a	0.094a	0.713 ± 0.031b

**Table 5:** Effect of Sb on the AsA, DHA, and ascorbate pool (AsA+DHA) contents and the AsA/DHA ratio, the GSH, GSSG, and glutathione pool (GSH+GSSG) contents and the GSH/GSSG ratio, in *D. viscosa*. The data are from 10 independent experiments, each carried out in triplicate (different letters indicate significant differences at *p* < 0.05, Mann–Whitney U-test).

Treatments	AsA (nmol g^−1^ FW)		DHA (nmol g^−1^ FW)		AsA + DHA (nmol g^−1^ FW)		AsA/DHA	
	Roots	Leaves	Roots	Leaves	Roots	Leaves	Roots	Leaves
Control	76.86 ± 7.63a	185.30 ± 15.61b	308.23 ± 18.57a	1827.49 ± 129.10a	385.06 ± 21.14a	2012.72 ± 92.75a	0.249 ± 0.030a	0.101 ± 0.009c
0.5 mM Sb	70.30 ± 8.53a	210.21 ± 10.17a	339.24± 28.13a	1288.89 ± 52.30b	409.54 ± 32.17a	1499.10 ± 105.11b	0.210 ± 0.024a	0.164 ± 0.009b
1.0 mM Sb	64.55 ± 8.05a	213.97 ± 22.80a	243.41 ± 13.10b	1021.50 ± 100.13c	307.92 ± 18.50b	1237.47 ± 107.98c	0.264 ± 0.027a	0.224 ± 0.044a
**Treatments**	**GSH (nmol g^−1^ FW)**		**GSSG (nmol g^−1^ FW)**		**GSH + GSSG (nmol g^−1^ FW)**		**GSH/GSSG**	
	**roots**	**leaves**	**roots**	**leaves**	**roots**	**leaves**	**roots**	**leaves**
Control	2.81 ± 0.32a	5.01 ± 0.72a	0.34 ± 0.04a	10.10 ± 1.82a	3.15 ± 0.30a	15.11 ± 1.53a	8.36 ± 1.3a	0.492 ± 0.035a
0.5 mM Sb	0.90 ± 0.12b	3.48 ± 0.16b	0.23 ± 0.11b	10.25 ± 0.86a	1.13 ± 0.21b	13.73 ± 1.10ab	3.91 ± 0.8b	0.342 ± 0.014b
1.0 mM Sb	0.54 ± 0.23c	2.60 ± 0.29c	0.14 ± 0.08c	8.31 ± 0.49b	0.68 ± 0.24c	10.91 ± 0.57b	3.85 ± 0.9b	0.316 ± 0.044b

## Data Availability

Data is contained within the article.
